# Long Island Medical College

**Published:** 1867-12

**Authors:** 


### Long Island Medical College.

It will be observed by our advertisement sheet, that this institution will commence its *ninth* regular session on the first of March, 1868.

It will also be observed that this College has made changes in its organization. The Chair of Anatomy is filled by Professor C. L. Ford, who is well known as one of the best teachers in his department. Professor Foster Swift, of New York, takes the Chair of Obstetrics, and Diseases of Women and Children. Prof. Armor is transferred to the Chair of Theory and Practice of Medicine, Prof. Austin Flint retaining Chemical Medicine.

As thus constituted, with most of its old professors retained, this College offers a teaching force which cannot be surpassed, and will be an attraction to students who desire to attend a Spring Course of Lectures.

